# The Opportunities and Responsibilities of the Medical Man of To-Day

**Published:** 1897-07

**Authors:** H. W. Cummings

**Affiliations:** President Brazos Valley Medical Association


For the Texas Medical Journal.

### THE OPPORTUNITIES AND RESPONSIBILITIES OF THE MEDICAL MAN OF TO-DAY.

H. W. CUMMINGS, M. D.,
President Brazos Valley Medical Association.

Read at Third Semi-Annual Meeting, Cameron, Tex., May 11, 12, 1897.

GENTLEMEN:—It is with pride that I see the Brazos Valley Medical Assocition passing into its second year of usefulness. And while I feel that its youth has, to a certain extent limited its influence, I wish to assure you that age is not the only, nor the most important essential to its welfare.

As I retire from its presidency and hand over the responsibilities of its future to my worthy successor, I trust sincerely that each one of you will unite with me in offering him our support and aid in his efforts to further the interest and influence of the Association. I feel sure that if we, as physicians, do our duty, some day not distant there will be cause for those men who answered the call to meet in Hearne a year ago, to feel proud that they gave their support to the organization of such a society.

I do not feel able to stand before men so much older in medicine than myself, and attempt to advise them, but as your by-laws has made it the duty of the retiring president to give some kind of an address to the Association, I am going to choose this hour to say something to you on "The Opportunities and Responsibilities of the Medical Man of To-day."

History teaches the observant mind that great intellectual progress proceeds along different lines at different epochs. In the days of Greece this development was largely in the line of literary activity. This was the epoch of great dramas, great and profound thought in philosophy. Then lived the great writers whose peers are never found in the present century. Later on in the world's history, Rome, apart from the influence of her arms in overthrowing artificial barriers and welding scattered tribes into nations, contributed much to human progress by establishing the Roman laws upon which modern jurisprudence is largely based.

Without the proper administration of justice, and the protection of free thinking and free speech, it is impossible for the genius of man to develop.

Thus in the turbulence of the Middle Ages there was no incentive for the mind to develop along the lines of its advancement of the present day. We of to-day are reaping the benefits of the struggles of by-gone heroes; and the achievements of the present have been made possible by the development of those forces and conditions which we call civilization. This is truly the inventive age, for we have done more, seemingly, for the progress of the world within the past fifty years than was done in centuries while the civilized world was in its infancy, but we should not fail to see the difference in the advantages for scientific research of to-day and the times of our forefathers. Then we can appreciate what greater responsibility to humanity we have to bear.

In times past great discoveries have been numbered but few in a century, each made by men, so isolated by their genius, that their names still live in the world's history. The past has been the day of the individual, to-day we lose sight of the man, but gain in power on account of the wonderful increase of the workers. The day has been when on account of the few men who devoted their time to study and investigation, the progress of any science was exceedingly slow. But on account of the present facilities of communication and travel, their number has been increased, and they have been brought in closer contact with their fellow-workers. The printing press and telegraph have come to the aid of science, and rescued from oblivion scattered treasures of observation and experience from all parts of the globe. Informed of the results of scientific research and labor throughout the world the work of the individual is shortened and simplified. Co-operation has its place in science as well as in trade and commerce, and just as the men in the commercial world succeed by their co-operative work, constantly keeping in touch with the values in the market as they rise and fall, just so will medical men progress as they are constantly kept informed of the discoveries and achievements made by their fellow-physicians. Nor are we barred from such progress, for the medical journal of to-day, with its digest, its clinical reports and its original articles, is a school within itself. It compresses into a year of reading, experience, which a century ago would take a lifetime to obtain by individual study and work.

The power of the press is one of the greatest blessings to the medical science, as its truths cannot fail to fall on fruitful soil, for be assured that it is the reading man and not the mere money-getter who is likely to be the investigator and the advanced worker.

It is true that the human mind, and especially the scientific mind, is now more active than a century ago, but is not this activity largely the result of constant interchange of thought through the means of societies and journals?

Nothing promotes narrowness of mind and contracted mental vision, more than isolation, and men who continue to be satisfied, alone, with the truths reached by their own investigation and experience, never willing to give or take in the knowledge of medical research, may assure themselves that it will only be a short time until their fellow-men have long left them in the rear.

Admitting, however, the great importance of medical men keeping themselves in close touch with the outside world by reading the various articles of value found in the pages of our modern journals, I am not persuaded that this means alone is prepared to make us what we ought to be in our profession. But there is yet a far more important and valuable means of gaining this knowledge. Reading widens our knowledge, but only by personal contact with each other can we catch the stimulus to personal effort to reach after higher things. Thus there is an element in modern society that literature cannot supply, and that is the personal influence of the individual. The cold pages of literature can never put heart into a man, nor create enthusiasm. We gain knowledge from reading, enthusiasm from personal contact, and indeed it requires both to bring a man upon that high plain which qualifies him to be a physician. Without enthusiasm there can be no true science, and the noble art of medicine degenerates into a business, the physician becomes a mere money-getter. There is a stimulus in companionship which cannot be overrated, and the societies of to-day supply us with the means of acquiring this stimulus. Ours is the responsibility if, by our absence, we fail to take advantage of such an opportunity; ours is the responsibility if, attending, we fail to participate in the discussions of our fellow-workers.

Here I wish to suggest the lack of effort and personal interest on the part of many of our members, and to urge upon you the necessity of giving more thought and more time to the welfare of this organization. Remember that your whole duty as a physician is not done when you have rendered your service to your patrons, and the many unfortunates who are compelled to depend upon your charity for the "balm of Gilead," but there is an obligation to the profession, which is not carried out until you have given all you know to them; that more widely may be the benefits to human ills. Indeed, no man can successfully steer the course of your Association without the co-operation of each individual member. And while I have no complaint to make—for you certainly have tendered your services well—yet I desire to urge you to more quickly answer the call from your new president, as he asks for your assistance in his labor to promote the welfare of our Association, as well as the interest of scientific medicine in our State.

The march of events in our profession is rapid. You are familiar with many of the wonderful advancements in the medical science, and well know that unless we men of Texas unite in one long and earnest effort to promote a higher standard of medical education and medical work, we will not long stand in the ranks. We must move on. What is being done elsewhere can be done here. We cannot stay long behind without lagging hopelessly in the rear.

Fortunes built up on personal magnetism are becoming rarer and rarer. The people are becoming better educated, and will soon distinguish between the success which is dependent upon purely personal qualities and that which is rooted in scientific knowledge. Ours is the responsibility, as well the privilege of familiarizing ourselves with the improved methods of to-day. The true physician is a public servant, and that man who never looks beyond his own immediate necessities, who regards the medical profession as but a means of support, whose daily rounds is but a tread-mill to coin dollars, will never attain the full measure of usefulness of which he is capable. There is hardly a walk in life, for educated men, which does not offer greater advantages for the accumulation of property than does the profession of medicine. For its votaries, the science and art of healing have other and higher rewards. The ability to relieve distress and suffering, to arrest disease, to restore the dying to the arms of affection, the consciousness of well doing for others, a life well spent. Aside from the intellectual pleasure man enjoys when he is conscious of doing a high grade of work, there ought to be a consciousness of service to his fellow being. After all, there is little in life unless we can reflect that we have done some good in the world to our fellow man in helping to lift the load of misery which oppresses the human race. Every one recognizes that scientific men of to-day in many ways excel those who have worked before us when the advantages to study were not so good. Yet many of you remember the old time doctor, and with what confidence his people hailed his coming, as he rode astride his pill bags, ministering to their hearts' bereavements as well as their physical ills. So after all our work, he gave quite as much consolation to the dying and bereaved as we do with our many means of saving life. To-day we can see with what gratitude his work is remembered as we see the thought picture in the following epitaph:

"There's a gathering in the village that has never been outdone
Since the soldiers took their muskets to the war of sixty-one.
And a lot of lumber wagons near the church upon the hill,
And a crowd of country people, sundry dressed and very still.
Now each window is pre-empted by a dozen heads or more,
Now the spacious pews are crowded from the pulpit to the door,
For with coverlets of blackness on his portly figure spread
Lies the grim old country doctor in a massive oaken bed:

Lies the fierce old country doctor,
Lies the kind old country doctor,
Whom the populace considered with a mingled love and dread.

Maybe half the congregation, now of great or little worth,
Found this watcher waiting for them when they came upon the earth.
This undecorated soldier of a hard, unequal strife
Fought in many stubborn battles with the foes that sought their life.
In the night time or the day time he would rally brave and well,
Though the summer lark was fifing or the frozen lances fell,
Knowing if he won the battle they would praise their Maker's name,
Knowing if he lost the battle then the doctor was to blame.
'Twas the brave old virtuous doctor,
'Twas the good old family doctor,
'Twas the faithful country doctor, fighting stoutly all the same.

When so many pined in sickness, he had stood so strongly by,
Half the people felt a notion that the doctor couldn't die.
They must slowly learn the lesson how to live from day to day
And have somewhat lost their bearings, now this landmark is away.
But perhaps it still is better that this busy life is done,
He has seen old views and patients disappearing one by one,
He has learned that death is master both of science and of art,
He has done his duty fairly, and has acted out his part,
And the strong old country doctor,
And the weak old country doctor,
Is entitled to a furlough for his brain and for his heart."

